# Sustainability of community health programme using community-based organizations: a challenge for stakeholders

**DOI:** 10.1186/s12913-023-09320-y

**Published:** 2023-05-04

**Authors:** F. M. Gisèle Donessouné, G. Olivier Sossa, Seni Kouanda

**Affiliations:** 1 University Joseph Kizerbo, Doctoral School of Health Sciences, Ouagadougou, Burkina Faso; 2 University Thomas Sankara, Ouagadougou, Burkina Faso; 3 Institute of Research of Health Sciences, Ouagadougou, Burkina Faso; 4 African Institut of Public Health, Ouagadougou, Burkina Faso

**Keywords:** Sustainability, Community organizations, TB programme

## Abstract

A community tuberculosis programme was implemented in Burkina Faso with funding from the Global Fund to fight HIV tuberculosis and malaria. Three years after the end of the funding, it seemed necessary to examine what remains of this innovative programme, which used civil society organizations for implementation. The objective of this study was to assess the factors that influence the capacity for sustainability and the level of sustainability of the programme.

**Methods** The case study approach was used to retrospectively identify the factors that influence the capacity for sustainability and the level of sustainability of the programme. Semi structured interviews were conducted with key informants. The data were analysed according to the theoretical frameworks of Schell and Pluye. Data was analysed using NVivo 12.

**Results** Strong support at the national level enabled the acquisition of funding for this programme, while the instability of financial resources was a drawback to sustainability. The lack of leadership of some associations did not allow the continuation of activities after the end of the funding. The irregularity of funding and the failure to conduct a final evaluation of the programme were the weakest links in the sustainability of the programme. Thus, the analysis of our data showed precarious sustainability at the time of the study.

**Conclusion** A well-designed community programme with a strong capacity for sustainability is not necessarily maintained after funding is withdrawn. The implementation of a community programme must ensure that it is integrated into the existing organizational system. The objectives and operating rules of the host structures must be in line with those of the programme to be implemented.

## Introduction

The implementation of development projects and/or programmes is confronted with the issue of sustainability, especially in the health sector. Sustainability remains a challenge in developing countries, which face budgetary constraints [1]. Maintaining the benefits of programmes for as long as possible is a necessity after funding ends [2]. Programme sustainability is a complex process with many challenges [3-6]. Programmes that are effectively implemented are likely to fail when their funding, planning, or training ends [2, 7]. Only 40–60% of programmes survive after the end of initial funding [2, 3, 8-10]. However, the goal of a programme is to contribute to sustainable development; therefore, to achieve their goals, community programmes should be continued as long as possible [9]. The start-up costs of a programme are considerable, so it is important to maintain the programme to achieve the desired impact. Early withdrawal would lead to a loss of confidence in communities. To date, the vast majority of research in the field of implementation science has focused more on issues of adoption and initial implementation of innovations, leaving aside their long-term fate [11, 12].

The sustainability of new programmes is influenced by many different factors [2, 13-16]. These factors can increase or inhibit the sustainability of a programme. By identifying these factors, managers can better control them to make programmes sustainable. Organizational capacity is the most important factor in ensuring sustainability. The existence of resources to effectively manage the programme and its activities is paramount. Partnerships between the programme and the communities help to strengthen the sustainability capacity of a programme. The fit between community needs and programme goals can predict sustainability [9]. Sustainability factors are classified into three categories: factors related to programme characteristics, factors related to the organizational context and factors related to the community environment [9].

Community-based programmes are an important part of the service delivery system in health promotion, but little is known about how these programmes are sustained [13]. Strong political support, building effective community partnerships, good communication, adapting the programme to local contexts and building organizational capacity in resource-limited settings have been seen as the main facilitating factors. However, unstable funding sources, a lack of evidence to prove programme effectiveness and a lack of strategic planning for implementation over time have been found to prevent the sustainability of many community programmes [17, 18]. To promote the sustainability of programmes, it is necessary that these factors be well identified and understood. This allows for a clearer conceptual understanding of how a sustainable programme is built from the start. While sustainability is a desired outcome of effective implementation, there is little research-based evidence available in this area in Africa, particularly in Burkina Faso (BF) [17]. Additionally, while several studies have examined initial implementation efforts, less research has been conducted to determine what happens beyond funding. Currently, the question of what happens to beneficiaries of health interventions when donor funding for implementation expires and how to measure this still persists [13, 18]. This is even more true in Africa, and more specifically in Burkina Faso, where, despite several rounds of external aid funding, little is known about sustainability. Budgetary reductions in external aid to resource-limited countries require actors to understand how to effectively allocate financial resources with a stronger focus on sustainability. If there are sustainability issues within the health system in general, then how do less organized structures such as community organizations fare? There are few studies that focus on the sustainability of complex innovations.^12,18.^ This fact calls to mind a community-based tuberculosis control programme that was implemented by community organizations under the supervision of the Support Programme for the Associative and Community(PAMAC), whose funding has ended. Although the Global Fund (GF) has suggested that recipient countries take sustainability into account in their project design, there is little or no research available on the sustainability of GF-funded community programmes. Efforts are being made in collaboration with governments, nongovernmental organizations (NGOs), civil society organizations (CSOs), the private sector and donor agencies to deliver effective and efficient health interventions, but sustainability issues are reducing the impact of these interventions^18^. What remains of this tuberculosis programme after three (3) years of funding ended? What were the factors that determined the programme’s level of sustainability? These are questions that our research sought to answer. Therefore, the objective of this study was to analyse the factors that influence the capacity for sustainability of a community tuberculosis (TB) programme implemented by PAMAC in BF. In addition, we aimed to assess the level of sustainability achieved by the programme at the time of the study.

## Methodology

### Description of the intervention

The community tuberculosis programme was financed by the GF. The community health workers (CHWs) were from Burkinabe civil society organizations (CSOs). CSOs are groups of volunteers whose members work in the health field and share a common religious practice (religious and customary), a professional link (traditional practitioners/therapists), the same experience of living with the disease (associations of former TB patients) or the same ideology (associations working in all areas of development: health, environment) [19]. This community programme differs from the community-based approach, which uses community health workers who are individuals chosen by their communities and are linked to district health services (Ministry of Health) [20]. A total of 22 associations in the five districts are involved in the programme. Two (2) members from each association have been trained to perform the activities. The package of offered activities is as follows:

- 1. Prevention activities: awareness-raising, information, education and communication, and behaviour change communication. These activities are used to increase the knowledge of the community and change behaviour.
- 2. Diagnosis activities: screening and contact tracing.

Identifying symptomatic individuals through active case funding. This strategy is used to systematically search for possible TB cases in communities. Presumptive TB cases are brought to the diagnosis and treatment centre (DTC) in health centres.

- 3. Treatment adherence support, home care and support. Community volunteers visit positive cases to supervise the use of anti-TB drugs.

These activities aim to provide TB diagnosis and treatment services to communities.

The following terms are used to refer to trained association members who are responsible for carrying out community activities: community health workers, community volunteers and animators. The implementing associations are supervised by two sub recipients (SR), namely, SR1: BURCASO and SR2: URCB. The Support Programme for the Associative and Community (PAMAC) is the Principal Recipient (PR) for GF funding. It is the financial management unit and supervises the SRs.

### Type of study

The current study was a retrospective case study. The community TB programme was the case, and we analysed it at two levels, namely, the capacity for sustainability and the level of sustainability achieved at the time of the study.

### Theoretical and conceptual framework

Several terms are used to refer to the concept of sustainability, including survival, continuation, maintenance, institutionalization, incorporation, integration and routinization. In our approach, we used the term sustainability, which refers to the viability of a programme after its initial funding period. In our case, sustainability refers to the continuation of the programme or part of the programme within the community organization [2, 21, 22]. 

In addition, we used the theory of organizational development to understand the institutionalization of change in organizations. This theory refers to how an organization maintains its knowledge such that it is available when needed [23] .

### Capacity of sustainability

We used Sarah Schell's framework, which was developed through an extensive literature review and concept mapping [4]. This framework is appropriate for our case study because it allows us to question public health programmes, including community programmes. The elements of this framework are as follows:

- 1. Political support

Political support influences whether the internal/external political environment is favourable to funding and to the acceptance of the programme.

- 2. Funding stability

The programme exists in a favourable state economic climate, the programme implements policies to ensure sustainable funding, and the programme is funded by a variety of sources.

- 3. Partnerships

Interaction/connection between the community and the programme.

- 4. Organizational capacity

This includes the internal support and resources needed to effectively manage the programme.

- 5. Programme evaluation

This system monitors and evaluates data related to the processes and results associated with programme activities is in place.

- 6. Programme adaptation

The capacity of the programme to be adapted to the local context.

- 7. Communication

This stage focuses on the existence of an established communication strategy with stakeholders and the public about the programme.

- 8. Impact on public health

This element assesses the programme's effect on the behaviour change, attitudes, and perceptions of beneficiaries.

- 9. Strategic planning

This stage involves questioning the project development process and the definition of objectives and strategies.

### Level of sustainability achieved during the study

To measure the level of sustainability, we used the theory of organizational routines. According to the authors of this approach, the level of sustainability can only be appreciated through the presence or absence of organizational routines [2, 14, 21].

Organizational routines are practically defined by the presence of four essential elements:

- Memory (financial, human, material and other)
- Adaptation (to context, effects, barriers)
- Rules (supervision, planning, tasks, regulation).
- Values (objectives, symbols, rituals, jargon)

When innovation is an integral part of the procedures and habits of an organization, it is said to be routine. Routines are embedded in organizations as the memory of actions or procedures shared by actors [14]. In our evaluative approach, we verified whether organizational routines (memory, adaptation, rules and values) exist in the community organizations three (3) years after the end of the GF TB community component funding. We assessed the level of sustainability of the community programme within each CSO. We supposed that within a given structure, the following would exist:

- zero sustainability: when there are no routine activities and therefore no organizational memory after the end of the programme.
- precarious sustainability: when there is an informal continuation of activities in the organization.
- high sustainability: when programme activities are an integral part of the organization.

**Fig. 1:**
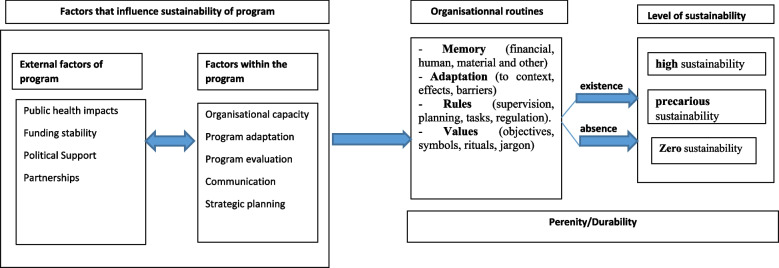
Conceptual framework for community TB programme sustainability

Figure 1 shows the link between the two frameworks.

### Data collection

#### Sampling and participants

The nature of our study led us to opt for purposive sampling to select the study participants. Purposive sampling was used for the individual interviews (IDIs). To include a wide range of views and experiences with the implementation of the intervention, all groups of key informants were selected. The choice of the central region was made to ensure the presence of a large number of key informants after the end of the programme. We chose to include all five (5) districts in the selected survey area. To ensure the representativeness of each group of actors, we systematically included all health facilities and members of associations that had implemented the intervention in the following health facilities: Bogodogo, Boulmiougou, Kossodo, Paul VI, and Samandin. We invited actors with knowledge of and experience with the implementation of the community TB programme to participate in the interviews. The interviews were conducted until the saturation point was reached.

### Data collection

Qualitative data were collected through in-depth interviews (IDIs) with key informants involved in the programme’s implementation. The interviews were conducted in French or Mooré (local language). We also collected information from programme documentation (activity reports, progress reports) to triangulate information. Data from interviews and information from programme reports were the main sources of data.

### Data collection tools

Two types of tools were used to collect the data: 1) data extraction grids for the reports and 2) interview guides. Five (5) different semi structured interview guides were used depending on the participants’ profile: (1) the SR informants, (1) the PR, (1) the Minitry of Health(MOH), (1) the Associations, and (1) the Diagnosis and Treatment Center (staff.)

Themes of discussion focused on the implementation process, implementing actors, programme funding, monitoring and evaluation, and the continuation of programme activities after funding ended.

### Data analysis

A deductive thematic analysis was used. We first proceeded by transcribing the interviews exhaustively in text format in Word software and then using NVivo version 12. Then, we structured our study corpus. The conceptual frameworks of Schell and Pluye guided our analysis. Through the coding process, we linked the verbatim passages to the themes and subthemes from the conceptual framework.

### Ethical considerations

We obtained ethics approval from the National Ethics Committee for Health Research No. 2017–4-41 40 of Burkina Faso on 3 May 2017. Before each interview, participants were informed of the purpose of the study, that their participation was voluntary, and that they could stop the interview at any time and for any reason. Informed oral consent was also obtained from all study participants. We assured he participants of anonymity and confidentiality before conducting the interviews. Any reference that identified the respondents was removed during the recording and analysis of the data.

## Results

The results are presented according to the nine (9) domains of Schell's framework [4].

### Enabling factors for the sustainability of community tuberculosis programmes

#### Political support

- Strong government commitment to community involvement

All interviewees recognized that policy-makers strongly support the involvement of associations in the fight against TB. This support is reflected in the development and adoption of strategies for CSO involvement in TB control.

"… it is undeniable that the political authority supports the participation of associations in the fight against tuberculosis…”. MoH official.

### Partnership

#### Population participation in community activities

The community participated in the awareness-raising activities carried out by the associations.

“When we saw an increase in attendance at our health centre, we were sure that the day before, there was an awareness-raising activity conducted by the associations.” Nurse Bogodogo.

#### Involvement of community leaders

Traditional healers sensitize patients who come to them. Patients with coughs are systematically taken to the health centre.

"…we played a very important role in this programme…we worked well with the health services who educated the population, and we drove those with coughs to the health centres." Traditional healer.

### Organizational capacity

#### Capacity building of community organizations

All the respondents said that this programme trained the members of the associations and equipped them with computers and vehicles.

"… There was a necessity to truly strengthen the associations at all levels… to provide them with equipment, human resources and training." NTP Program manager.

On the other hand, there were differing views in terms of the true capacity that was actually built.

In summary, I found that there wasn’t a true capacity building, but rather material support, which didn’t build the associations for sustained growth." NTP Monitoring Evaluator.

### Programme evaluation

#### Setting up a community-based monitoring and evaluation (ME) system

An ME plan was developed for the monitoring of indicators. A mid-term evaluation carried out at the end of the second year permitted the revision of the strategies and data collection tools. This evaluation recommended a fiduciary agency to strengthen financial management.

"The programme was so well conducted that we stopped, evaluated and corrected what did not work. SR_3_ programme manager.

The mid-term evaluation allowed for the replacement of some activities with other more promising ones.

"… The evaluation recommended moving away from mass awareness-raising activities to outreach activities, such as door-to-door educational talks". SR_3_ programme manager.

### Programme adaptation

#### Adapting the programme to the local context

Adaptations were made to ensure effective implementation. As the activity reports and interviews show, awareness-raising, referral and monitoring and evaluation activities were modified to fit with context.

"The PR understood us.… we adapted from time to time… otherwise, it was not easy". SR_1_ data manager.

### Communication

There was effective communication between health workers and community actors at the central level. This communication was appreciated at the central level by the programme managers. According to the respondents, effective collaboration between the NTP (Ministry of Health) and the CSO enabled the establishment of good permanent communication.

"… we were always invited to the periodic meetings, whether at the regional or district level… not to mention the informal communication with the health workers." Association leader 2.

### Barriers to the sustainability of the community programme

### Partnership

#### No commitment from local authorities

Partnerships with local authorities were absent.

"The local authorities were not fully involved… I feel like they were passive in this programme…" CSO leader.

The community programme in our study did not sufficiently establish a cross-sector partnership.

"… I find that the community programme was limited to health actors only… in my opinion, it was necessary to open up to not only city councils and governorates but also the private sector… this could even be a channel for obtaining other funding…" an NTP executive.

### Stability of funding

#### Irregularity of funding

Funding stability has been the weakest link in this programme.

"… During the programme, we were never sure of the arrival of financial resources… discontinuity, delays… sometimes a whole quarter passed without funding…" SR_2_ coordinator.

#### Unavailability of local funding

The government of Burkina Faso has not provided any budget for the community component according to most of the surveys, and there is a lack of consideration of the community component by the government.

"The health services truly see the work done by the associations… it is a shame that all the substantial funding comes only from external partners. When they leave, what will happen to the community activities? Association leader 1.

### Organizational capacity

#### Institutional strength/capacities

The lack of sufficient and qualified staff has led several associations to contract with civil servants.

"At first we did not understand how other SRs could deliver satisfactory results; these associations used local officials to prepare reports and even carry out other activities. SR_3_ programme manager.

The results show a weak organizational capacity of most civil society organizations.

"Many of these associations have nothing serious… they are empty shells ‘represented’ by the president and some members of his family" MoH official.

This organizational deficit very quickly created governance problems within these associations that led to interpersonal conflicts. The results indicate that project staff members were paid (including different perks and benefits) through the GF. This team constituted a unit separate from the rest of the staff in the association. It was therefore expected that the project staff, rather than the association staff, would support the continuation of the programme.

"All the staff were there for the community TB Global Fund project… and were paid by it.… there were no leaders since they were all gone as soon as the funding ended…" Association Member 2.

### Communication

#### Communication failure at the peripheral level

According to respondents, there was a communication gap with the target audience (community and beneficiaries). Indeed, respondents from the peripheral level structures that were supposed to implement the operational activities stated that they did not always have access to strategic information.

*"In the future, we have to make sure that the association members receive the information... it is not easy... it is not necessarily those who carry out the activities who go to the information meetings*.”

### Strategic planning

The planning was considered participatory at the level of the project designers and managers, but the actors in charge of the operational implementation did not feel involved in the process.

"We made sure that everything was participatory… that the communities were represented…" MoH official.

"We were just asked to come and listen to what they had to say in Ouaga; otherwise, we did not feel involved in the implementation stages." Association leader.

#### No sustainability planning

With regard to planning resource mobilization, the actors were more concerned with trying to meet the conditions (financial management in accordance with the GF's policy, achieving the objectives) than with thinking about alternative sources of financial resources:

"… We had no choice but to satisfy the GF… it was very time-consuming, so we did not think… we thought that by respecting their conditions of implementation, we would obtain new funding…” SR2 programme officer.

### Programme evaluation

A final evaluation of the community TB programme was not carried out, which some respondents noted as a weakness. It was necessary to know if the programme was worth maintaining and, if so, which aspects to maintain.

"… The programme ended abruptly; we were expecting a real final evaluation to know what worked and what did not… but that was not possible" BS manager.

### Results of the level of sustainability

#### Existence of an organizational memory (financial, human, material and other)

GF funding helped to strengthen the capacity of PR and SR in terms of human and material resources.

*“... We cannot complain; the recruitments were done, and all the BS team members were university graduates..."* BS monitoring officer

However, at the level of the associations, the strengthening was insufficient

"… What I did not understand was that in contrast, the associations were not as well off as they should have been… there was a shortage of qualified human resources and insufficient numbers…" ME officer.

Although the associations retained some of the elements of organizational memory (in terms of materials acquired, personnel recruited), it was found that since the end of funding, most associations have used these resources for activities other than the community TB programme.

The results show that TB control activities were carried out informally in some associations.

"… We do not do TB anymore; we did not get any money, it is only the materials that remain… but they are not used." Association manager.

"Human resources have left; some have joined the public service, and others have sought work or are now working for associations that have received funding…" Association manager.

Another source of funding was not found during the time period of our survey to replace the funding that ended.

"… Since the end of the funding, we have stopped everything… there are no other sources of funding" Coordinator of an association.

#### Existence of guidelines

Guidelines and procedures were established for all programme actors. Management tools (procedural manuals, specifications, agreements, training participant manuals) contained the management rules and procedures approved by the GF.

The intervention was aligned with the values of the health system organizations; for example, the community objectives were defined taking into account the district objectives.

"Associations plan activities according to our objectives…so, we even do that together." DTC manager.

The common values that the associations and districts shared were sealed by an agreement signed with the regional health director. Additionally, the existence of common objectives made it possible to develop a symbol, that is, a referral card. Such cards represent the tangible measure of the effectiveness of the intervention in the health centre.

"… According to the colour of the cards, the health workers know automatically which association made the referral…in fact, we communicate through the cards." Animator SR 1.

The link between the existence of funding and the continuation of this programme within the associations was established.

*"*… *Without funding, how can we talk about the sustainability of the programme?"*

The members of the programme team left the associations.

"We could not keep the people. How were we going to motivate them? They all left in search of their daily bread." President of association X.

The difficulties of these associations in retaining the staff recruited by the programme made it difficult to maintain the”organizational memory” that is essential for the sustainability of TB activities in the organization.

"… People think we're still doing TB there; when they cough, they come to our headquarters, or we direct them to the ones that were selected for the current grant… we do not get any money…" Animator X.

"We do not carry out any more activities, eh… except that often there are people who come to inform us that someone in their environment is coughing. We refer them, and we tell them that we are no longer in the programme… we no longer have funding… how do you expect us to work?” Animator Y.

Organizational routines were put in place during the implementation, from the design to the end of the programme funding. As the results show, no association was able to maintain these organizational routines. Sustainability was therefore precarious, as no community structure 'routinized' the programme activities. Only informal referrals of TB suspects are made at people's request.

## Discussion

The objective of our study was to analyse the capacity for sustainability and the level of sustainability of a community tuberculosis programme implemented by PAMAC in BF. Using the framework method, we were able to identify factors that influenced the sustainability of this programme [4, 24]. There are two types of such factors, namely, those that promoted sustainability and those that jeopardized the programme's sustainability.The results show that political support, partnership, adaptation, and communication were the factors that promoted sustainability. Political support allowed the country to benefit from GF funding for the implementation of community-based activities, enabling material, human and financial resources to be deployed for the implementation of this programme. However, the level of support was not strong enough to allow the country to mobilize local resources to make up for the end of financial support from the GF. In principle, the mobilization of local resources could have facilitated sustainability.

The organizational capacity of the associations, the irregularity of funding and the failure to conduct a final evaluation of the programme were the weakest links in the sustainability of our programme. Regarding the level of sustainability 3 years after the end of GF funding, the data synthesis indicates that the community programme at the time of data collection had precarious sustainability. The main challenge was the understanding of the concept of sustainability by programme designers and managers. Sustainability was only seen from the funding side. Thus, there was confusion between the sustainability of funding and the sustainability of the programme itself. The analysis of our results clearly indicates that the concept of sustainability is very strongly associated with the presence of financial resources according to most respondents. In this perception, the sustainability of the programme is guaranteed if the funding is renewed. Sustainability is therefore perceived by the actors as the continuation of external aid (Global Fund financing), and any structure that no longer receives these resources does not envisage moving towards sustainability. One of the perverse effects of external funding is that it conditions actors to always expect to be paid for their involvement in the implementation of activities. This situation contributed to compromising the sustainability of our programme [14, 17]. This starting point has led to a certain focus on economic and financial aspects and has led to sustainability being treated more often than not from an economic perspective.

This starting point has led to a certain focus on economic and financial aspects and has resulted in sustainability being treated most often from an economic perspective.

However, sustainability is not only about financial viability. In the same way, donors tend to see sustainability as a financial issue. Beneficiaries in turn perceive this perception and react accordingly [25]. However, while financial sustainability is often the 'weak link', i.e., the point at which an activity or structure collapses when the support project ends, it is only one of the conditions; other dimensions need to be taken into account to achieve it [5]. Our analyses suggest the need to clarify this notion at the programme design stage to ensure that all aspects of sustainability are taken into account.

Despite the significant investments in terms of material, human and financial resources granted to community organizations, the results show a low level of sustainability of the community programme. No community activities are officially conducted in these associations. Moreover, the few activities that have been carried out are no longer capitalized on, despite the existence of capitalization tools. These activities still exist but have no routinization characteristics. This is easily understood since we are dealing with community organizations that function with recruited human resources (presence conditioned by remuneration); to ensure the presence and involvement of personnel, their remuneration must be guaranteed [26]. Another challenge therefore exists with regard to the associations that have had difficulty keeping these actors without providing financial motivation. Additionally, the major consequence of this "turnover" of personnel has been the loss of "organizational memory". It is understandable why there was no emergence of a champion to carry the future of the project (sustainability planning, reflection on alternative sources of funding, on the continuity of activities and services). The greatest obstacle to sustainability was the weakness of the associations' organizational capacity [1]; most association structures do not have the necessary resources to guarantee the management of the programme after GF funding stops. No financial resource planning has been done to mobilize financial resources to continue the activities, yet sustainability is very strongly linked to the stability of financial resources. With only one source of funding, the structures were not necessarily able to maintain the programme [27]. It is now known that sustainability increases more when there are multiple sources of funding or when other funding strategies are considered. Otherwise, it is difficult to maintain activities [8].

From this perspective, sustaining programme activities without special external funding is more likely to occur if the programme components are integrated into organizational processes [5, 9, 14]. However, in our case, not all association structures have a formal organizational framework. Missions and goals are oriented to the funding opportunities present in the external environment. We can safely say that these structures are fighting for their own survival. It may be unrealistic to expect a particular programme to be sustained if the host organization is struggling for financial survival or implementing other complex interventions [28]. Our results reflect programme characteristics that have been demonstrated by several studies to be conducive to sustainability [27]. However, this programme has shown a low level of sustainability, and few, if any, activities have been "formally in the routine" of the associations. In the absence of financial resources, the sustainability of interventions implemented by individual providers is likely to be strongly influenced by their motivation to continue the new practice [28]. The integration of a programme into a structure must fit into the existing organizational system and not be a one-time project. Attention is again drawn to the sustainability of vertical programmes [9, 14]. The lack of organizational risk-taking forces organizations to manage the programme autonomously without integrating it into their usual organizational circuit; it is therefore considered a one-time project that can be terminated at the slightest constraint [9, 17].

Our analysis therefore suggests that the characteristics of the host organization (organizational environment, management capacity) are more important in determining the sustainability of a programme it hosts. This assertion differs from that of some authors who have concluded that the sustainability of a community programme depends more on the quality of the programme [9, 13, 21].

One difficulty often encountered during the programme closure process is that attention is usually focused on opportunities to find other sources of funding, which means that an important aspect is overlooked, namely, the final evaluation. Several studies have made the same observation [17]. However, this evaluation is crucial in that it shows in terms of sustainability which aspects should be continued and which should be abandoned or readjusted. However, it could be that there is no interest in continuing the intervention. In this case, the programme is abandoned because there is no longer any need to continue a programme that is not worth the effort [2, 3].

When the continuation of programmes is far beyond the means of the community, the end of external funding does not represent the beginning of a sustainability phase but instead leads to the end of the sustainability process. Thus, although there is a proven and expressed demand by the community, the associations express their inability to carry out the activity although it requires little or no financial resources to do so. In this case, we believe that the presence of the donor in the immediate environment would prevent sustainability. The donor having renewed the programme with associations other than those previously involved makes them passive and demanding of their reintegration into the financing circuit. Lafond, 1995, as quoted by Calhoun A [29], stated that "too many" external resources can be detrimental to sustainability.

Finally, organizations are concerned with meeting donor-imposed implementation conditions rather than adapting to the local context. Organizations may also feel pressured to adapt their activities to donor priorities, leading to donor influence on priority setting and timeframes for implementation, funding, and reporting, as well as feedback on what could be supported, how, and by whom. This reduces the chances of sustainability when donor funding stops.

## Limitations of our study

The first limitation of the current study is that we could not include direct beneficiaries to assess sustainability in the community. The decision to exclude direct beneficiaries was based on the fact that another grant was being implemented. Including the beneficiaries would have introduced confounding bias, which we sought to avoid. Additionally, because this subsidy still exists in the immediate environment of the associations involved in the study, there was a risk that the respondents would tell us what would appear to be the norm.

The second limitation is that the choice of health region did not allow us to have a truly holistic view of the level of sustainability of the entire programme. Finally, given the time that had elapsed (3 years) between programme closure and the survey implementation, there may be memory bias.

For future research, it would be interesting to highlight the degree of sustainability in relation to the programme components (i.e., which programme components have the highest level of sustainability and why). This could be taken into account in the future design of this type of programme.

## Conclusion

The community TB programme analysed herein was designed with the potential for sustainability. Using the Schell framework, we were able to identify the factors that determined the sustainability of this programme. Despite the investments made in community-based organizations, there was little sustainability. The weak organizational capacity of the implementation structures could explain this situation. The implementation of a community programme should ensure that it is integrated into the usual organizational system and, above all, that the objectives and operating rules of the host structures are in line with those of the programme to be implemented.

Programmes that have been given a high degree of sustainability are more resilient to unexpected events such as budget cuts and infrastructure changes. To ensure that the benefits of a programme are maintained for as long as possible, managers, designers, and all other stakeholders must plan activities with a focus on sustainability.

## Data Availability

The datasets generated and/or analysed in the course of this study are not publicly available due to [the fact that the same database is being used for the writing of other articles of my thesis. However, if necessary I can share with interested readers] but are available from the corresponding author upon reasonable request.

## Abbreviation

DTC: Diagnosis and treatment of tuberculosis center
PAMAC: Support Programme for the Associative and Community
BURCASO: Burkinabe Council of Community Development Organisations
URCB: Union of Religious and Customary Leaders of Burkina Faso
